# Modeling of Bridging Law for Bundled Aramid Fiber-Reinforced Cementitious Composite and its Adaptability in Crack Width Evaluation

**DOI:** 10.3390/ma14010179

**Published:** 2021-01-02

**Authors:** Daiki Sunaga, Takumi Koba, Toshiyuki Kanakubo

**Affiliations:** Department of Engineering Mechanics and Energy, University of Tsukuba, 1 Chome Tennodai, Ibaraki 305-8573, Japan; s2020844@s.tsukuba.ac.jp (T.K.); kanakubo@kz.tsukuba.ac.jp (T.K.)

**Keywords:** FRCC, aramid fiber, bundled fiber, bridging law, fiber orientation, bilinear model, uniaxial tension test, crack width

## Abstract

Tensile performance of fiber-reinforced cementitious composite (FRCC) after first cracking is characterized by fiber-bridging stress–crack width relationships called bridging law. The bridging law can be calculated by an integral calculus of forces carried by individual fibers, considering the fiber orientation. The objective of this study was to propose a simplified model of bridging law for bundled aramid fiber, considering fiber orientation for the practical use. By using the pullout characteristic of bundled aramid fiber obtained in the previous study, the bridging laws were calculated for various cases of fiber orientation. The calculated results were expressed by a bilinear model, and each characteristic point is expressed by the function of fiber-orientation intensity. After that, uniaxial tension tests of steel reinforced aramid-FRCC prism specimens were conducted to obtain the crack-opening behavior and confirm the adaptability of the modeled bridging laws in crack-width evaluation. The experimental parameters are cross-sectional dimensions of specimens and volume fraction of fiber. The test results are compared with the theoretical curves calculated by using the modeled bridging law and show good agreements in each parameter.

## 1. Introduction

Fiber-reinforced cementitious composite (FRCC) is cementitious material reinforced with short discrete fibers showing ductile behavior of composite, especially in tensile and bending stress. In the past several decades, various types of FRCCs, such as ductile fiber-reinforced cementitious composite (DFRCC) [1], strain hardening cement composite (SHCC) [2], and engineered cementitious composite (ECC) [3], have been studied by lots of researchers. DFRCC shows a deflection hardening behavior and multiple cracking behavior under bending field. SHCC and ECC show pseudo-strain-hardening behavior and multiple-fine-cracking behavior under uniaxial tension. These types of FRCCs have been applied for the actual structures such as walls, beams, slabs and decks, tunnel linings, etc. [2,3]. It has been expected to extend the application of FRCCs with additional values for resilience and sustainability of structures.

The high tensile performance of FRCC is brought by the bridging effect of fibers across cracks after initial cracking of matrix. For that reason, fiber-bridging stress–crack width relationships called bridging law have been studied by lots of researchers to evaluate tensile characteristics of FRCC. In the case of FRCCs without showing multiple cracking behavior, bridging law can be obtained from a uniaxial tension test [4,5], or indirectly from the bending test of a prism specimen [6]. In recent years, even in SHCC showing multiple-cracking behavior, some methodologies to measure the crack width of a single crack have been introduced by some researchers [7,8]. On the other hand, Li et al. introduced theoretical solution of bridging law based on the micromechanics [9]. Since the bridging performance is strongly affected by the fiber types and dimensions, the pullout behavior of an individual fiber from cementitious matrix has been investigated, to reflect these factors on bridging law. Several researchers have conducted pullout tests for various types and dimensions of fibers, e.g., steel fiber [10,11], nylon and polypropylene (PP) fibers [12], aramid, and polyvinyl alcohol (PVA) and polyethylene (PE) fibers [13].

The bridging law can be given by an integral calculus of forces carried by the individual bridging fibers, considering the fiber orientation and distribution [14]. Since the bridging performance of FRCC is influenced by the casting method and the dimensions of the specimens [15], it is essential to evaluate the effect of the fiber orientation and distribution. For that reason, evaluation methods of fiber orientation, considering fabrication methods of specimens, have been studied by several researchers [16,17]. The authors have also studied the influence of casting direction on fiber orientation and tensile characteristics of PVA-FRCC through visualization simulation, using water glass solution [18]. To evaluate the fiber orientation distribution quantitatively, a probability density function (PDF), using an elliptic function (elliptic distribution), was introduced in that study. The bridging law was calculated by using the elliptic distribution, considering casting direction. The calculated bridging laws showed good agreements with the uniaxial tension test results of FRCC specimens fabricated by horizontal and vertical casting. In addition, the influence of casting method and specimen dimensions on fiber orientation of PVA-FRCC was also studied by the authors [19,20].

The bridging law has a potential to be the base in the evaluation of the structural performance of FRCC members [21,22]. However, it is not convenient to adapt the calculated bridging law directly for various types of structural members because the bridging law remarkably varies by the fiber orientation. Attempting an easy use of the bridging law, the authors have modeled the bridging law of PVA-FRCC as the tri-linear model, which is characterized by fiber orientation [23]. It is considered that this model of bridging law makes it easier to evaluate structural performance of FRCC members. In fact, the authors have conducted the evaluation of bending characteristics [23] and crack width in steel-reinforced PVA-FRCC members [24] by using the model. Providing the models of bridging laws not only for PVA-FRCC but also for various FRCCs using another type of fiber helps to make the most of the potential of FRCC in the practical use.

Aramid fiber is one of the polymeric fibers that shows high tensile strength, durability, and heat and chemical resistance. A few researchers have studied about FRCC reinforced with short discrete aramid fibers [25,26,27]. Since a commercially provided single aramid fiber has a small diameter of 12 µm, the high bond strength between the cementitious matrix cannot be expected [13]. Although PVA fiber shows a good bond in the matrix because of the alcohol group in PVA molecule [28], other types of polymer fibers do not generate large bond resistance because of the smooth surface. In the case of steel fiber, the contrivances, such as a twisted shape, hooked end, deformed surface, etc., are generally applied to make good bond performance [29]. For these reasons, the authors have been focusing on the bundled aramid fiber, which is made with a bundling of original yarn of aramid fiber to improve the bond performance between the matrices. The authors have conducted the pullout test of an individual bundled aramid fiber from the cementitious matrix [30]. The pullout characteristic was expressed by a bilinear model based on the test results. Bridging law can be calculated by using the model, and the calculated results showed good agreements with the experimental results obtained by the uniaxial tension test of aramid-FRCC. However, as previously mentioned, the bridging law should be modeled by a simple form, considering the fiber orientation, to utilize aramid-FRCC effectively in various types of structural members.

The objective of this study was to propose a simplified model of bridging law of bundled aramid-FRCC, considering fiber orientation. By using the pullout characteristic of bundled aramid fiber obtained from the previous study [30], the bridging laws are calculated by assuming various cases of fiber orientation. The calculated results are expressed by a bilinear model, and each characteristic point is shown by the function of fiber-orientation intensity, which gives the main parameter of the elliptic distribution [18]. The modeled bridging laws are adapted to evaluate the crack width in steel-reinforced aramid-FRCC members. The uniaxial tension test of aramid-FRCC prism specimens with rebar is conducted to measure the crack-opening behavior experimentally and the test results are compared with the theoretical ones [24], in which the bridging law is included.

## 2. Calculation of Bridging Law and Modeling 

### 2.1. Calculation of Bridging Law

**Figure 1:**
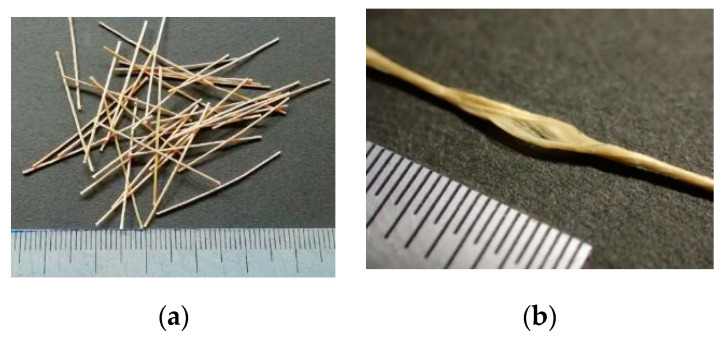
Visual appearance of used aramid fiber: (**a**) chopped aramid fibers for mixing fiber-reinforced cementitious composite (FRCC) and (**b**) Condition of bundling of yarns.

The aramid fiber that was the focus of this study was bundled fiber with a nominal diameter of 500 µm, as precisely the same as that studied in the previous study [30]. Figure 1 shows the visual appearance of the fiber. The original yarns with a nominal diameter of 12 µm (Technora, TEIJIN, Arnhem, The Netherlands [31]) are twisted to form a thick individual fiber and sized not to unravel in the matrix. According to the manufacturer test results, the tensile strength and elastic modulus of the yarn are 3432 MPa and 73 GPa, respectively. Chopped fibers with a length of 30 mm are used for mixing FRCC.

**Figure 2:**
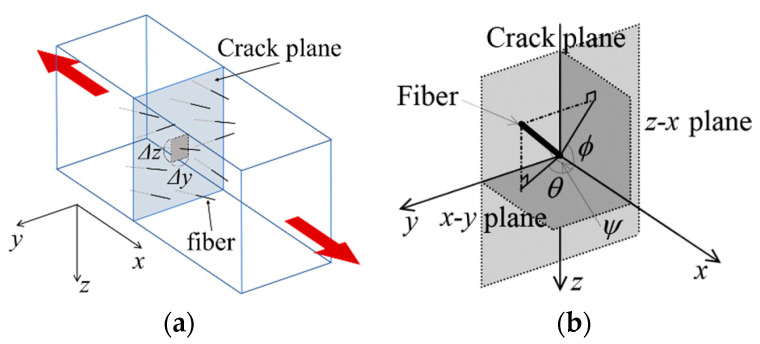
Schematic drawing for the calculation of bridging law: (**a**) fibers bridging through crack plane and (**b**) definition of fiber inclination angle.

The bridging law (bridging stress–crack width relationship) is calculated as similar with the previous study [30]. In a FRCC prism subjected to the uniaxial tension, fibers bridge through crack plane as shown in Figure 2a. Fibers are distributed in crack plane with various inclination angle. The pullout behavior and rupture strength of the individual fiber is affected by the fiber inclination angle that is defined as shown in Figure 2b. The angle, *ψ*, expresses the fiber inclination angle to *x*-axis, and angles, *θ* and *φ*, express the angle between *x*-axis and projected lines of the fiber to *x*–*y* plane and *z*–*x* plane, respectively. The bridging stress can be calculated by summation of forces carried by individual fibers bridging through crack plane considering PDF for fiber inclination angles and fiber centroidal location as given by Equation (1).

(1) $$\begin{array}{c} \sigma_{bridge}=\frac{P_{bridge}}{A_{m}}=\frac{V_{f}}{A_{f}}\cdot \underset{h}{\sum }\underset{j}{\sum }\underset{i}{\sum }P_{ij}\left(w,\psi \right)\cdot p_{xy}\left(\theta_{i}\right)\cdot p_{zx}\left(\Phi_{j}\right)\cdot p_{x}\left(y_{h},z_{h}\right)\cdot \Delta \theta \cdot \Delta \Phi \left(\Delta y\cdot \Delta z\right) \end{array}$$

where *σ_bridge_* = bridging stress, *P_bridge_* = bridging force, *A_m_* = cross-sectional area of specimen, *V_f_* = fiber volume fraction, *A_f_* = cross-sectional area of an individual fiber, *w* = crack width, *P*(*w*,*ψ*) = pullout load of an individual fiber, *p_xy_* = PDF (elliptic distribution) for fiber inclination angle in *x*–*y* plane, *p_zx_* = PDF (elliptic distribution) for fiber inclination angle in *z*–*x* plane, *p_x_* = PDF for fiber centroidal location (assumed to be constant), and Δ*y·*Δ*z* = area of infinitesimal element on crack plane.

The bilinear model proposed in the previous study [30] is adapted for the pullout load of an individual fiber, *P*(*w*, *ψ*). The elliptic distribution [18] is adopted for the PDF, *p_xy_* and *p_zx_*, for fiber inclination angles. The elliptic distribution is defined by two parameters; principal orientation angle, θ*_r_* (argument of one radius of elliptic function), and orientation intensity, *k* (ratio of the two radii of elliptic function). The fiber orientation can be expressed by these parameters. The random orientation is given by *k* = 1. Fibers tend to orient toward θ*_r_* when the value of *k* is larger than 1, while fibers tend to orient toward the perpendicular to θ*_r_* when the value of *k* is smaller than 1. The PDF for fiber centroidal location, *p_x_*, is set to constant assuming the uniform distribution of fibers along *x*-axis.

**Figure 3:**
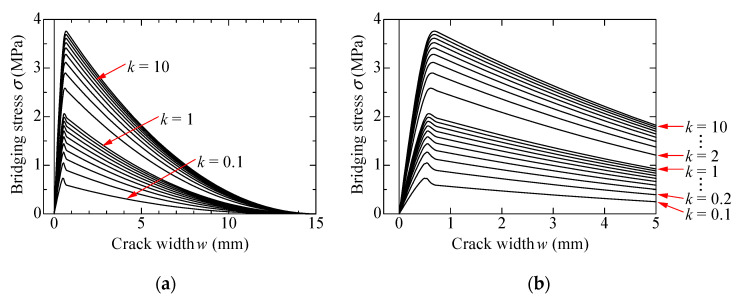
Calculation results of bridging law: (**a**) *w* = 0–15 mm; (**b**) *w* = 0–5 mm.

The calculated bridging laws for the orientation intensity *k* ranging from 0.1 to 10 are shown in Figure 3. The bridging laws shown in the figures are calculated with 0.1 intervals of *k* in the case of *k* < 1, and with 1 interval when k > 1. The left figure shows whole curves, and the right figure focuses on small ranges until *w* = 5 mm. The parameters adopted for the calculation are summarized in Table 1. Fiber volume fraction and principal orientation angle is set to 2% and 0°, respectively. The bridging stress in Figure 3 do not include the tensile stress carried by the matrix before cracking to exhibit the tensile stress due to only bridging force of fibers. Each curve in Figure 3 shows the maximum bridging stress at about *w* = 0.6 mm. After that, bridging stress decreases moderately with the increase of the crack width. This is because most of fibers do not rapture, and they are gradually pulled out from the matrix. Bridging stress becomes zero when the crack width reaches to 15 mm (half length of the fiber) because all fibers are completely pulled out from the matrix. On the other hand, by comparing each curve, the maximum bridging stress remarkably increases with the increase of the value of *k*. In other words, bridging stress becomes larger when the fibers strongly orient to the normal direction of the crack surface. The examples of the adaptability of the calculated bridging law with the uniaxial tension test results of aramid-FRCC by notched specimens can be found in the previous study [30]. The calculated results show good agreements with the experimental ones.

### 2.2. Modeling of Bridging Law

**Figure 4:**
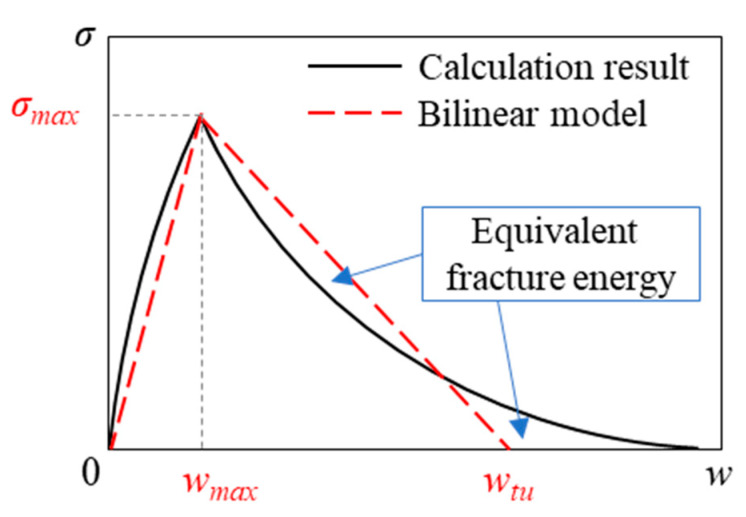
Bilinear model for bridging law.

**Figure 5:**
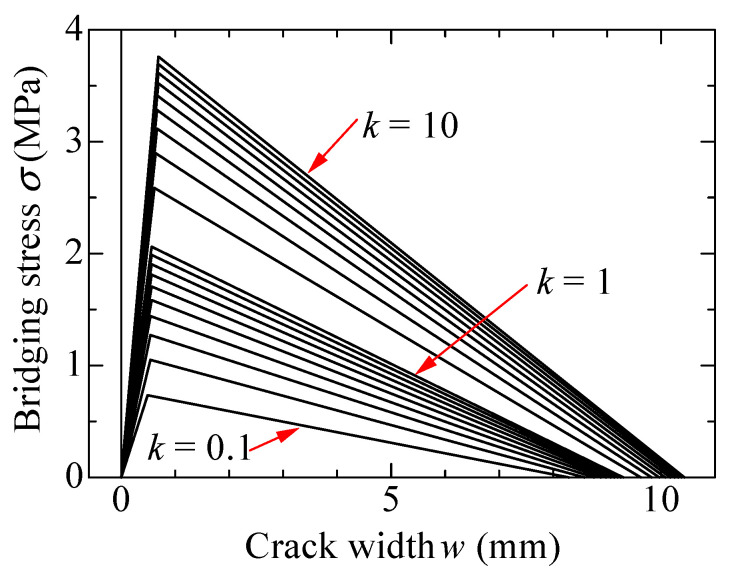
Modeled bridging law.

**Figure 6:**
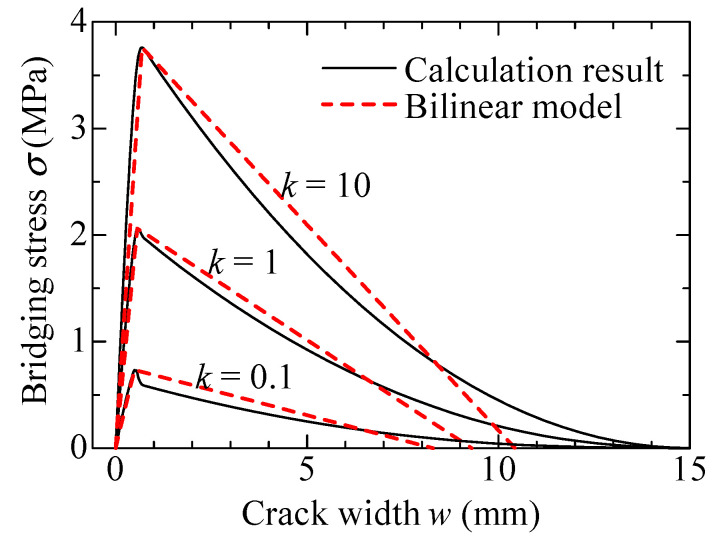
Comparison between the calculated bridging law and model.

The calculated bridging laws are modeled by simple forms that consider fiber orientation, to utilize them effectively in various types of structural members. From Figure 3, the bridging law is simply characterized by two regions, i.e., the curve until the maximum stress and softening branch. Therefore, the bridging law is expressed by a bilinear model, as shown in Figure 4. The model has three parameters: the maximum bridging stress, σ*_max_*; the crack width at maximum bridging stress, *w_max_*; and the crack width when bridging stress at zero, *w_tu_*. The values of σ*_max_* and *w_max_* of the model can be obtained directly from the calculation results. The value of *w_tu_* is determined to have an equivalent fracture energy with the calculated bridging law in the softening branch. The modeled bridging laws for each fiber orientation intensity, *k*, are shown in Figure 5. The comparison between the calculated bridging laws and the models for *k* = 0.1, 1, and 10 are shown in Figure 6. 

**Figure 7:**
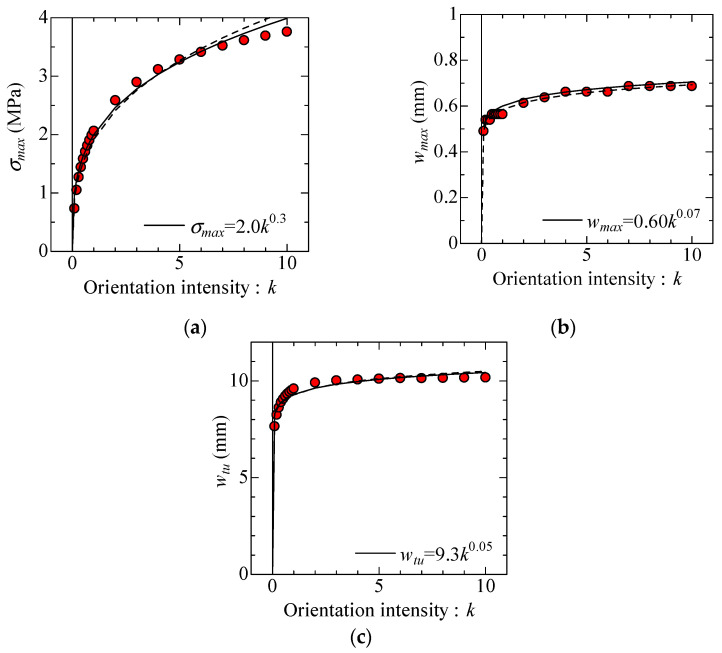
Relationship between each parameter of model and orientation intensity *k*: (**a**) σ*_max_*-*k* relationship, (**b**) *w_max_*-*k* relationship, and (**c**) *w_tu_*-*k* relationship.

The three parameters in the model are expressed as a function of the fiber orientation intensity, *k*, to simplify the modeling of bridging law. The relationships between the parameters and *k* are shown in Figure 7. The dotted lines in all figures exhibit the regression calculation results by the least square method. The solid lines exhibit the modified regression calculation result, to simplify the relational expression between each parameter and *k*, as given by Equations (2)–(4).

(2) $$\begin{array}{c} \sigma_{max}=2.0k^{0.3}\;\left(\mathrm{MPa}\right) \end{array}$$

(3) $$\begin{array}{c} w_{max}=0.60k^{0.07}\;\left(\mathrm{mm}\right) \end{array}$$

(4) $$\begin{array}{c} w_{tu}=9.3k^{0.05}\;\left(\mathrm{mm}\right) \end{array}$$

The characteristic points of bilinear model of bridging law (Figure 4) in each fiber orientation intensity, *k*, can be easily obtained by using these formulas.

## 3. Uniaxial Tension Test of Aramid-FRCC Specimens with Steel Rebar

### 3.1. Specimens and Used Materials

**Figure 8:**
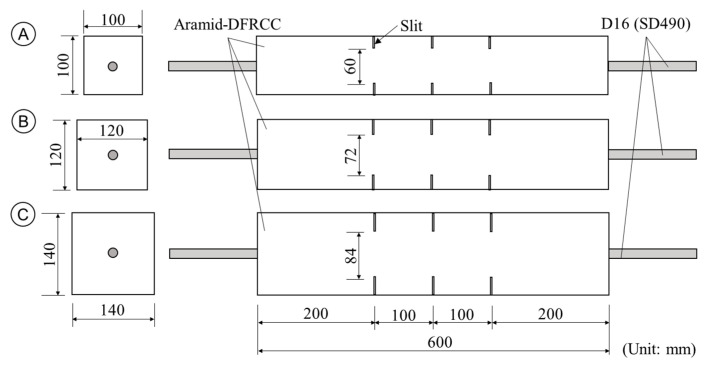
Dimension of specimens [24].

Uniaxial tension test of aramid-FRCC prism specimens with steel rebar is conducted to measure the crack-opening behavior experimentally. Figure 8 and Table 2 show the dimensions of specimens and the list of specimens, respectively. The specimens have same dimensions with those in the previous study by the authors [24], and the mixture proportions of the matrix are also the same as in the literature [30]. The No Fiber series specimens tested in the previous study by the authors [24] are focused on again, to contrast the test results in this study. The specimen is FRCC prism reinforced with a single steel rebar along the longitudinal direction. The prism has a square cross-section, and the length of the prism is 600 mm. Steel deformed rebar D16 is used for the reinforcement. The bundled aramid fiber previously mentioned is used for FRCC. The test parameters are cross-sectional dimensions and volume fraction of fibers. The cross-section was 100 mm × 100 mm (A series), 120 mm × 120 mm (B series), and 140 mm × 140 mm (C series). To ensure the position of cracking, slits were installed on both specimen sides, at 100 mm spacing. The depth of slit was varied in each series of specimens so that the cross-sectional area at the slit was 60% of the full sectional area. These slits were installed after demolding the specimen by using a concrete cutter, so as not to disturb the fiber orientation. The volume fraction of aramid fiber was set to 1% and 2%, and three specimens were fabricated in each combination of parameters. 

**Figure 9:**
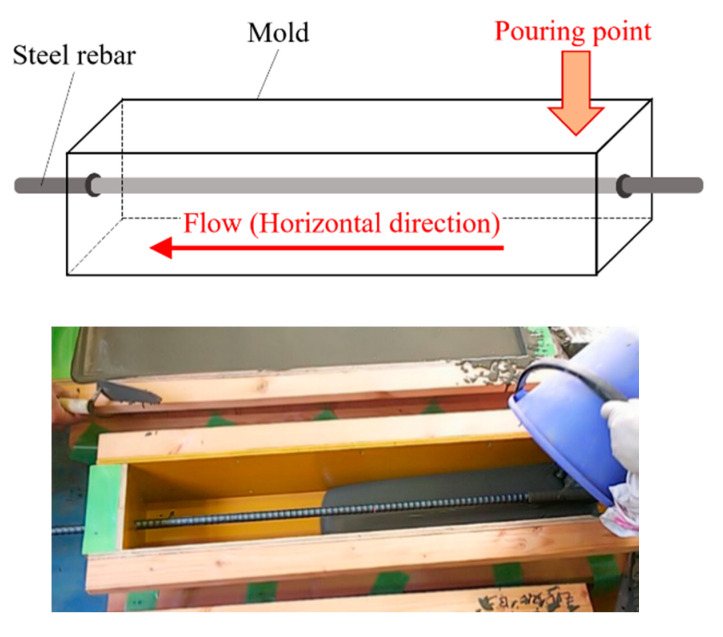
Casting method of FRCC.

Table 3 lists the mixture proportion and mechanical properties of FRCC. The mixture proportion is precisely same as the previous study conducted by the authors [30]. Since the fresh FRCC shows self-compacting characteristics, FRCC was filled into the mold by pouring from one longitudinal end of the mold, as shown in Figure 9, similar with the previous study [20], while making sure not to disturb the fiber orientation. The compressive strength and elastic modulus of FRCC, as shown in Table 3, were obtained from compression test of Φ100 mm × 200 mm cylinder test pieces. Table 4 lists the mechanical properties of reinforcing bar. A steel deformed rebar with diameter of 16 mm (nominal value) was used for the reinforcement. These values are obtained from the tension test of steel rebar in the previous study [24].

### 3.2. Loading and Measurements

**Figure 10:**
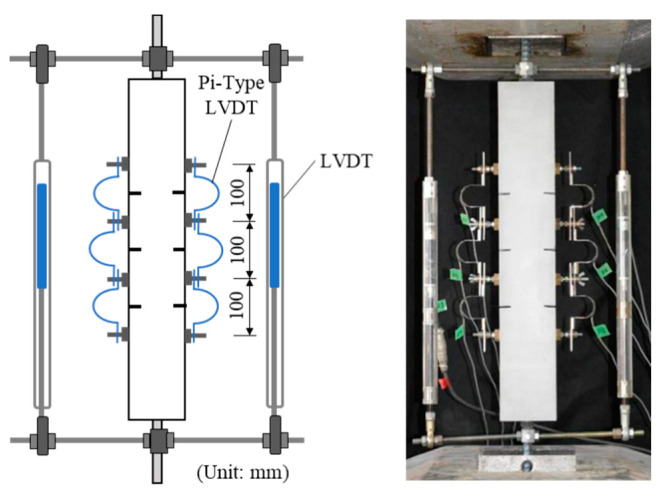
Setup of loading and measurement [24].

Figure 10 shows the setup of loading and measurement. Uniaxial monotonic tension loading is conducted under the controlled displacement using universal testing machine. The total deformation was measured by two linear variable displacement transducers (LVDTs) to confirm the yielding of steel rebar. Crack width at each slit position was measured by Pi-type LVDTs arranged at 100 mm spacing on both side of the specimens. The criteria of defining crack width are explained in detail in Section 3.3. Visible crack observations were recorded in each loading and measurement step. These loading and measurement methods are consistent with the previous study [24].

### 3.3. Test Results

**Figure 11:**
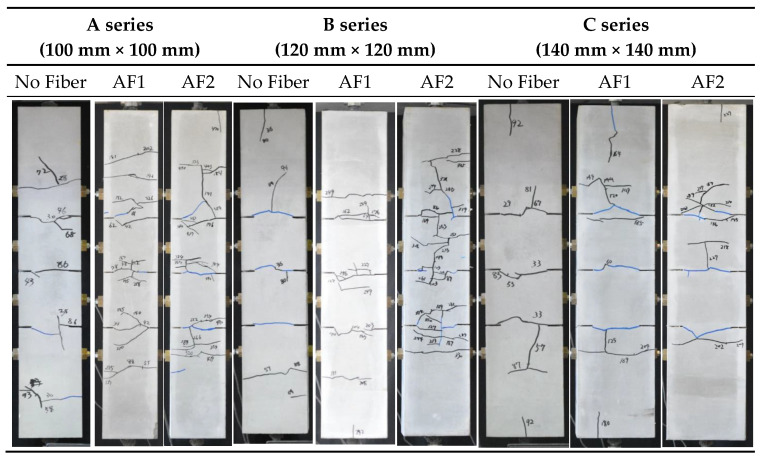
Examples of crack patterns after steel rebar yielding [24].

Figure 11 shows the examples of crack patterns after yielding of steel rebar. The specimens with fewer cracks in the longitudinal direction were selected as the examples in each parameter. Cracking at slit position was observed before the yielding of steel rebar in every specimen. However, branched cracks at the slit positions, or another crack at the no-slit position, were observed in many specimens. The total number of cracks increased with the decrease of the cross-sectional area and increase of the fiber volume fraction.

**Figure 12:**
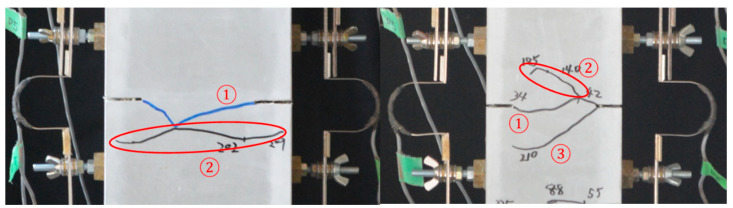
Examples of cracking process ( 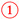 first crack, 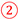 second crack, and 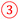 third crack).

In the case of a single crack at slit position, the crack width is obtained by averaging two values measured by Pi-type LVDTs on both sides of specimen ignoring the elastic deformation of FRCC. After the second crack was observed in one measurement region as shown in Figure 12, the measured data at that region were excluded from the evaluation, so that the measured crack width corresponds to a single crack.

**Figure 13:**
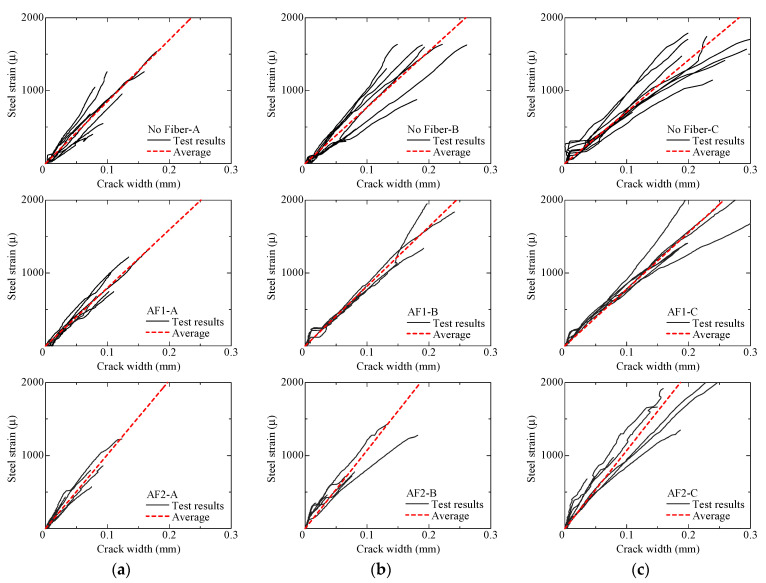
Steel strain–crack width relationship: (**a**) A series; (**b**) B series; (**c**) C series.

Figure 13 shows the steel strain–crack width relationships. The steel strain is calculated from the measured tensile load by using the elastic modulus of reinforcing bar previously shown in Table 4. The crack width of these curves increases just after the beginning of the loading due to the deformation of FRCC in the measurement region. The average line of test results is shown in Figure 13 as dotted line to compare the crack-opening behavior. The average line is obtained by averaging the slopes of approximate straight lines (*y* = *a*·x) of experimental curves in each parameter.

The slope of the average lines is larger in AF2 specimens compared with AF1 specimens in the same series of cross-section. Since the fiber bridging effect increases with increasing of fiber volume fraction, crack width tends to be smaller in the specimens with many fibers. This tendency is consistent with the previous studies [24,32,33]. However, no large difference is observed between No Fiber and AF1 specimens. It is considered that the number of fibers bridging through the crack is too small to control the crack opening in AF1 specimens.

On the other hand, the slope of average lines decreases with the increase of cross-sectional dimensions, when comparing among No Fiber-A, -B, and -C specimens. Crack width tends to be larger because the number of cracks decreases with increasing of cross-sectional area as mentioned before. In the case of AF1 and AF2 specimens, the difference of cross-sectional area affects less on the crack opening, compared with the case of No Fiber specimens because of the bridging effect of fibers. This tendency is also consistent with the previous studies [24].

## 4. Adaptability of Modeled Bridging Laws in Crack Width Evaluation

In this section, to verify the adaptability of modeled bridging law in Section 2 in crack width evaluation of steel reinforced aramid-FRCC, the theoretical curve of steel strain–crack width relationship is calculated by using the crack width prediction formula, in which bridging law is included and compared with the test results obtained in Section 3.

### 4.1. Theoretical Curve of Steel Strain–Crack Width Relationship

In the case of uniaxial tension test of steel reinforced FRCC prism, the theoretical curve of steel strain–crack width relationship is given by Equation (5) [24]. This formula is led by considering bond interaction between FRCC and rebar, fiber bridging effect at crack surface and cracking condition of FRCC.

(5) $$\begin{array}{c} \begin{array}{c} \varepsilon_{s\left(LOAD\right)}=\frac{k_{bo}\varphi_{s}}{8A_{c}\left\{\sigma_{cr}-\sigma_{br}\left(w_{cr}\right)\right\}}w_{cr}{}^{2}+\frac{1+np}{2npE_{c}}\left\{\sigma_{cr}+\sigma_{br}\left(w_{cr}\right)\right\} \end{array} \end{array}$$

where *ε_s(LOAD)_* = strain of rebar at loaded end, *w_cr_* = crack width, *σ_br_* (*w_cr_*) = bridging law (function of crack width, *w_cr_*), *σ_cr_* = cracking strength of FRCC, *k_bo_* = bond stiffness between matrix and rebar, *φ_s_* = perimeter of rebar, *A_c_* = cross-sectional area of FRCC, *A_s_* = cross-sectional area of rebar, *E_c_* = elastic modulus of FRCC, *E_s_ =* elastic modulus of rebar, *p* = reinforcement ratio (=*A_s_*/*A_c_*), and *n* = ratio of elastic modulus (=*E_s_*/*E_c_*).

**Figure 14:**
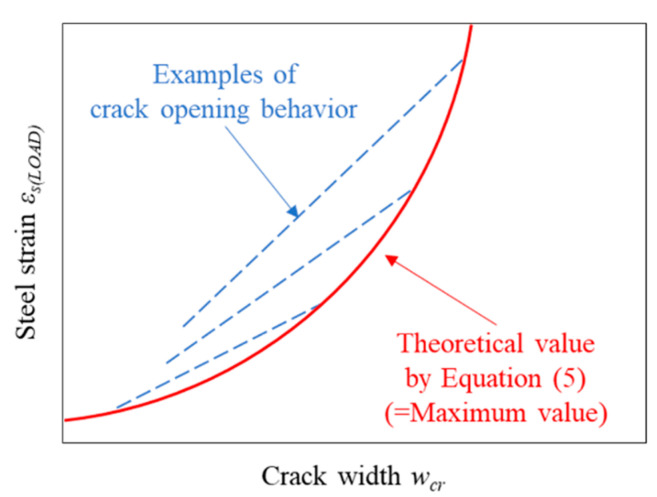
Steel strain–crack width relationship given by Equation (5).

Note that Equation (5) does not give the crack-opening behavior of a single crack but gives the possible maximum value of crack width at arbitrary strain of rebar as shown in Figure 14. This is because Equation (5) is led by using the condition that a new crack generates. This is based on the assumption that the generation of a new crack increases the number of cracks and decreases the crack width of each crack; hence, the crack width of a certain crack does not become larger than the theoretical value calculated by Equation (5).

Table 5 shows the parameters adapted for the theoretical formula, Equation (5). The perimeter and cross-sectional area of steel rebar correspond to the nominal values. The elastic modulus of FRCC and steel rebar is obtained from material tests described in Section 3.

On the other hand, since it is difficult to obtain the cracking strength of FRCC directly from the material test, the value is assumed from the test results of uniaxial tension test. According to the experimental curves in Figure 13, it can be confirmed that the crack width increases rapidly at the early stage of the loading in No Fiber-C, AF1-B, and AF2-B specimens. It can be assumed that the cracks start opening at these steel strains (No Fiber-C, 324μ; AF1-B, 239μ; and AF2-B, 318μ). These values are converted to the tensile loads by using cross-sectional area and elastic modulus of steel rebar. The converted tensile loads are divided by the cross-sectional area of specimens at slit position (B series, 120 mm × 72 mm; C series, 140 mm × 84 mm), and the cracking strength of No Fiber, AF1, and AF2 series specimens is calculated as shown in Table 5. The same value from the previous study [24] for bond stiffness between the steel rebar and FRCC is used.

The modeled bridging law of aramid-FRCC in Section 2 is adapted for Equation (5). The fiber orientation intensity, *k*, for the model is decided based on the results of previous study in which the size effect on fiber orientation of FRCC [20] has been investigated. 

In that study, four-point bending test was conducted for three different dimensions of PVA-FRCC prism specimens with 40 mm × 40 mm, 100 mm × 100 mm, and 180 mm × 280 mm in cross-section. The section analysis was performed by using bridging law of PVA fiber, considering several cases of fiber orientation intensity, *k*. The test results of 100 mm × 100 mm cross-section specimens showed the best agreement with the analytical results in bending strength by assuming *k* = 1. 

**Figure 15:**
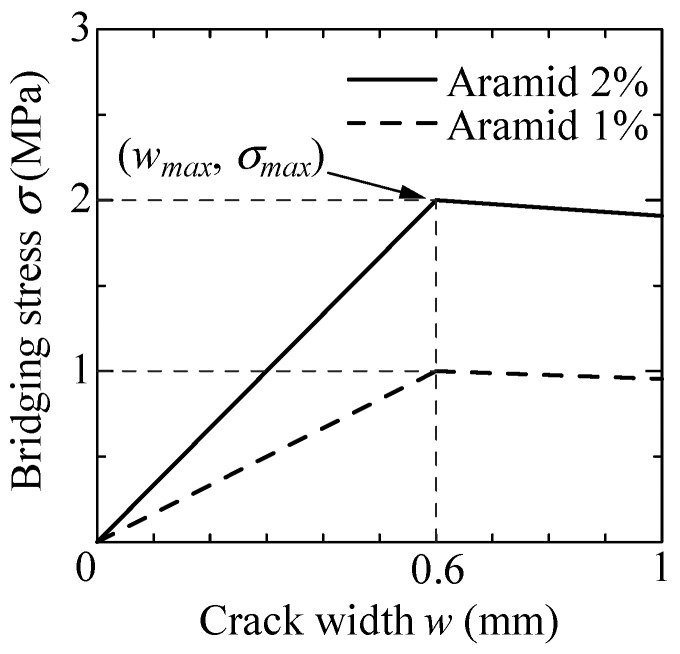
Bridging law model of aramid-FRCC used for evaluation.

For these reasons, *k* = 1 is also adapted for Equations (2)–(4), and the model shown in Figure 15 is used for the evaluation. In FRCC specimens with fiber volume fraction of 1%, the bridging stress is considered half as much as that of 2%, as shown by the dotted line in Figure 15. When the bridging law is substituted for the theoretical formula, bridging stress is reduced as 0.6 times, which corresponds to the ratio of cross-sectional area at slit position to the whole section, in order to take the absence of bridging fibers at slit into account.

### 4.2. Comparison between Theoretical Curve and Test Results

**Figure 16:**
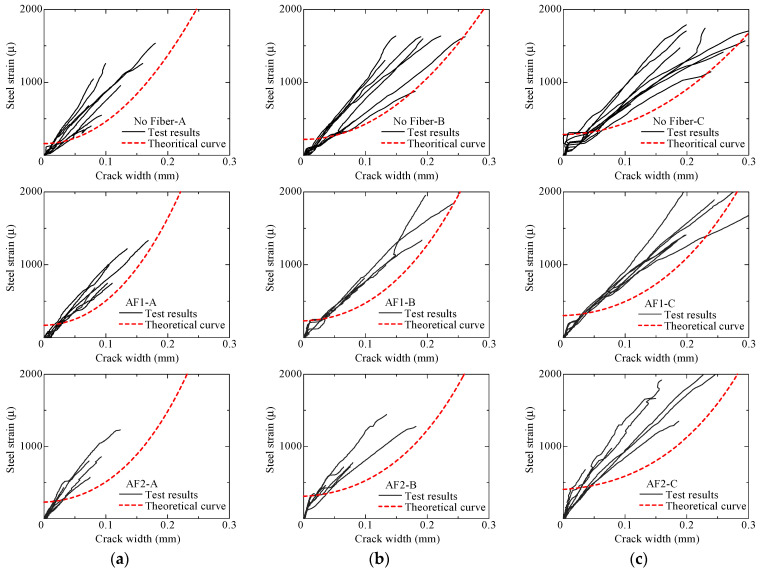
Comparison of theoretical curve and test result: (**a**) A series; (**b**) B series; (**c**) C series.

Figure 16 shows the comparison of theoretical curve and test result. The theoretical curve shows the possible maximum value of crack width, while the curves of test results show the crack opening behavior of each crack, as mentioned before. By comparing the theoretical curves among No Fiber, AF1, and AF2 specimens in the same series of sectional dimensions, the crack width at the same steel strain is smaller in specimens with larger volume fraction of fibers. 

The curves of test results in most of the specimens are located inside the smaller crack-width region than the theoretical curves. It can be concluded that the theoretical curves show good agreements with the test results. It is considered that crack-width evaluation for steel-reinforced aramid-FRCC is also adaptable, using the modeled bridging law. 

In this study, crack width evaluation was performed as the example of actual application of proposed bridging law model. Although the model was simplified by the function of fiber orientation intensity, *k*, only one case of *k* = 1 was experimentally verified. It is considered that the fabrications of specimens controlling fiber orientation is not easy. The results of the previous study that discusses the size effect on bending characteristics of FRCC [20] show the possibility to control the fiber orientation by dimensions of the specimens. In uniaxial tension test specimens adopted in this study, however, smaller sectional-area specimens become to show unexpected cracks in axial direction of the specimen, larger sectional-area specimens may show few cracks that satisfy the enough level of crack width evaluation before rebar yielding. Further experiments with various types of aramid-FRCC specimens, considering these viewpoints, are necessary to investigate the generality of the models in the future research. 

## 5. Conclusions

Based on the modeling of bridging law for aramid-FRCC and crack-width evaluation through experimental program, the following conclusions are drawn:

1. To propose the simplified model of bridging law of bundled aramid-FRCC, the bridging law is calculated by assuming various cases of fiber orientation and expressed as bilinear model. The characteristic points of the model are given by the function of fiber orientation intensity.
2. The uniaxial tension test of aramid-FRCC specimens with steel rebar is conducted, and crack-opening behavior is measured experimentally. The crack width tends to be smaller in AF2 (fiber volume fraction of 2%) specimens, compared with No Fiber and AF1 (that of 1%) specimens.
3. The theoretical curves of steel strain–crack width relationships are calculated by using the modeled bridging law. The calculated curves show good agreements with the test results in each parameter.

## Figures and Tables

**Table 1:** Parameters for bridging law calculation.

Parameter		Input
Fiber Volume Fraction, *V_f_* (%)		2.0
Length of Fiber, *l_f_* (mm)		30
Diameter of Fiber, *d_f_* (mm)		0.5
Apparent Rupture Strength of Fiber, *σ_fu_* (MPa) [30]		σ*_fu_* = 1080 · *e* ^−0.667*ψ*^
Bilinear Model [30]	Maximum Pullout Load, *P_max_* (N)	*P*_max_ = 7.47 · *l_b_*
	Crack Width at *P_max_*, *w_max_* (mm)	*W*_max_ = 0.13 · *l_b_*^0.64^

Notation: ψ = fiber inclination angle to *x*-axis (rad.); *l_b_* = embedded length of fiber (mm).

**Table 2:** List of specimens.

Type	Common Factor	Cross-Section (Section at Slit)	Volume Fraction of Fibers
No Fiber-A [24]	Length: 600 mm Number of slits: 6 Spacing of slits: 100 mm Steel rebar: D16 (SD490) Fiber: Bundled aramid	100 mm × 100 mm (100 mm × 60 mm)	-
AF1-A			1.0%
AF2-A			2.0%
No Fiber-B [24]		120 mm × 120 mm (120 mm ×72 mm)	-
AF1-B			1.0%
AF2-B			2.0%
No Fiber-C [24]		140 mm × 140 mm (140 mm × 84 mm)	-
AF1-C			1.0%
AF2-C			2.0%

**Table 3:** Mixture proportion and mechanical properties of FRCC.

Type	Unit Weight (kg/m^3^)					Compressive Strength (MPa)	Elastic Modulus (GPa)
	Water	Cement	Sand	Fly Ash	Aramid Fiber		
No Fiber [24]	380	678	484	291	0	52.5	18.1
AF1					13.9	48.2	18.1
AF2					27.8	47.5	16.4

**Table 4:** Mechanical properties of steel rebar.

Type	Yield Strength (MPa)	Yield Strain (μ)	Elastic Modulus (GPa)	Tensile Strength (MPa)
D16 (SD490)	516	2604	198	709

**Table 5:** Parameters adapted for theoretical formula.

				No Fiber [24]	AF1	AF2	Remarks
Steel Rebar	Perimeter	*φ_s_*	Mm	50			Nominal Value
	Cross-Sectional Area	*A_s_*	mm^2^	198.6			
	Elastic Modulus	*E_s_*	GPa	198			Material Test
FRCC	Cross-Sectional Area	*A_c_*	mm^2^	A:100^2^, B:120^2^, C:140^2^			–
	Elastic Modulus	*E_c_*	GPa	18.1	18.1	16.4	Material Test
	Cracking Strength	*σ_cr_*	Mpa	1.03	1.09	1.45	Tension Test
Bond Stiffness		*k_bo_*	N/mm^3^	50			[24]

## Data Availability

Data sharing is not applicable to this article.
